# Research on health impacts of chemical contaminants in food 

**DOI:** 10.2471/BLT.21.287532

**Published:** 2022-03-01

**Authors:** Samira Choudhury, Antonieta Medina-Lara, Richard Smith, Nicholas Daniel

**Affiliations:** a College of Medicine and Health, University of Exeter, St Luke's Campus, Heavitree Road, Exeter, EX1 2LU, England.; b Analytics Division, Food Standards Agency, London, England.

One of the most fundamental determinants of our health is the food we eat. While research on the quantity and quality of food has increased, mostly because of rising obesity levels worldwide,^1^ far less research on food safety is available. Although some focus on acute diseases associated with microbiological foodborne pathogens exists,^2^ the risks associated with chemical contaminants are severely under-researched.

Chemical contamination may occur at any point of the various stages of processing, packaging, transportation and storage of food.^3^ Chemical contamination may also be the result of environmental contamination, as in the case of toxic metals, polychlorinated biphenyls and dioxins, or through the intentional use of chemicals such as pesticides, veterinary medicinal products and food contact materials.^3^ Effects of chemical contaminants in food are associated with acute episodes with a single exposure (for example gastrointestinal illness caused by paralytic shellfish poisoning) or chronic due to repeated long-term exposure (such as liver cancer due to chronic exposure to mycotoxins).^4^^–^^8^

Estimating the burden of foodborne disease from chemical contaminants presents several challenges, related to this breadth of contamination and its multiple effects. First, quantifying how many chemicals and toxins enter the food supply and at what point of the supply chain is difficult.^9^

Second, estimating the burden of some chemicals such as fish toxins and persistent organic pollutants may require epidemiological studies for extrapolating biomonitoring data and combining with relevant toxicity data.

Third, the health effects caused by chemicals in foods such as aflatoxin, causing liver cancer, or lead, causing kidney cancer, may not be observable until years after exposure and require longitudinal studies, which are time consuming and expensive. 

Fourth, because health outcomes are multicausal, establishing the relationship between exposure to chemical contaminants in food and the development of disease is complicated. As a result, data associated with exposure to effect (that is, dose–response) are often limited or must be derived from in vitro or in vivo studies. Furthermore, toxicological risk assessments may not consider long-term exposure through foods and other sources and the linkage of exposure with the probability of adverse health effects. This link is crucial since consumers are constantly exposed to various chemical contaminants that may potentially result in synergistic effects. Indeed, one single chemical can exert multiple health effects. For instance, a chemical may be a reproductive toxin and a carcinogen.^10^

Fifth, chemical contaminants in food may only harm human health when exposed at dose levels that are high enough to cause adverse health effects.^10^ However, for some chemicals, unless there is a potential risk, identifying exposure level is not possible. These chemicals refer to environmental chemicals directly affecting the human genome but also some foodborne metals causing, for example, neurobehavioural effects or aplastic anaemia.^10^

These five challenges limit current studies seeking to quantify the health threats posed by chemical contaminants in food and have led the scientific community to place less emphasis on tackling them. The most recent global and regional disease burden estimates from arsenic, cadmium, lead and methylmercury^11^ highlight major limitations in this research base. For example, outcomes that reflect the relationships between lead and hypertension, and arsenic and heart disease, were not included. Similarly, these studies did not account for other estimates of seafood toxins, pesticide residues, food additives (sulphites, nitrites, etc.), mycotoxins other than aflatoxin, process contaminants (acrylamide, polycyclic aromatic hydrocarbons, etc.) and natural contaminants (aminoglycosides, aristolochic acid, etc.). All these elements may be important with respect to the impact of chemicals and toxins on the foodborne burden of disease.

The impact of chemical contamination in food is a neglected area of health research. We need estimates of the health burden to raise awareness of the effects of foodborne chemical contaminants in the public health community. We also need to engage with food safety stakeholders across countries to develop mitigation strategies and efficiently allocate resources for disease prevention. Doing so will require better surveillance data and adequate measures of exposure assessment to accurately reflect the current incidence of dietary exposures to chemical contaminants. Chemical-specific biomonitoring data to assess exposure as well as epidemiological data on other diseases associated with chemicals in food need to be developed. Confirmation of the cause of illness from chemical exposures and the application of formal epidemiological methods must be improved to quantify the baseline disease burden and associations with outcomes. These steps are critical to understanding and determining the true burden of foodborne disease from chemical contaminants, which is foundational knowledge in helping us tackle this largely hidden global health threat.
